# Effect of Perioperative Management on Outcome of Patients after Craniosynostosis Surgery

**Published:** 2017-01

**Authors:** Abdoljalil Kalantar Hormozi, Nastaran Mahdavi, Mohammad Mehdi Foroozanfar, Seyed Sajad Razavi, Razavi Mohajerani, Ahmad Eghbali, Amir Ali Mafi, Haleh Hashemzadeh, Alireza Mahdavi

**Affiliations:** 1 Department of Craniomaxillofacial Surgery, Mofid Hospital, Shahid Beheshti University of Medical Sciences, Tehran, Iran;; 2 Department of Pediatric Anaesthesiology, Mofid Hospital, Shahid Beheshti University of Medical Sciences, Tehran, Iran;; 3 Shahid Beheshti University of Medical Sciences, Tehran, Iran

**Keywords:** Craniosynostosis, Pediatric, Intensive care unit, Operation

## Abstract

**BACKGROUND:**

Craniosynostosis results from premature closure of one or more cranial sutures, leading to deformed calvaria and craniofacial skeleton at birth. Postoperative complications and outcome in intensive care unit (ICU) is related to surgical method and perioperative management. This study determined the perioperative risk factors, which affect outcome of patients after craniosynostosis surgery.

**METHODS:**

In a retrospective study, 178 patients with craniosynostosis who underwent primary cranial reconstruction were included. Postoperative complications following neurosurgical procedures including fever in ICU, level of consciousness, re-intubation, and blood, urine, and other cultures were also performed and their association with the main outcomes (length of ICU stay) were analyzed.

**RESULTS:**

Factors independently associated with a longer pediatric ICU stay were fever (OR=1.59, 95% CI=1.25-4.32; *p*=0.001), perioperative bleeding (OR=2.25, 95% CI=1.65-3.65; *p*=0.01), age (having surgery after the first 5 years) (OR=1.59, 95% CI=1.33-3.54, *p*=0.016) and infection (OR=2.17, 95% CI=1.83-7.46; *p*=0.002). Mean blood loss during surgery was significantly higher in patients whose duration of ICU was longer than 4 days compare to less than 4 day (*p*=0.026). Amount of bleeding significantly was correlated to duration of surgery (r=0.70, *p*=0.001) and patient’s age (r=0.23, *p*=0.44).

**CONCLUSION:**

perioperative management particularly blood loss could deteriorate patients outcome and length of stay in ICU and hospital. Infections in ICU could deteriorate outcomes.

## INTRODUCTION

Craniosynostosis is premature closure of one or more cranial sutures, leading to deformed calvaria at birth. The cranial vault is composed of a series of bony plates, of which conjunctions constitute the cranial sutures. In some situations, brain growth is retarded by prolonged restriction of the cranial vault secondary to fusion of the overlying sutures. The rate of isolated non-syndromic craniosynostosis in newborn population has been reported to be approximately 1 in 2000 to 3000 live births.^1^^,^^2^

Surgical outcome is significantly influenced by surgical techniques, perioperative events and post-operative complications.^3^^,^^4^ Therefore, perioperative period should be monitored for early diagnosis of any predictive complicating factors. The knowledge of these complications and poor outcomes is based on previous experiences, collection of data from peripoerative events and illustrations of predictive factors. Besides, there is always possibility of severe complications, which could compromise patients’ outcome.^4^

Post-operative complications of cranioplasty are divided into immediate and delayed. Immediate complications include blood loss, air embolism, and dural tear with CSF leak, infection, and respiratory disorders. Blood loss can be rapid and hemodynamically significant with tear of the venous sinuses or major cortical veins.^5^ Blood loss may be continued for 12–24 h following cranioplasty and monitoring in ICU is crucial.^6^ Infection is another major complication, which occurs in approximately 7% of patients undergoing craniofacial surgery. Clinicians should be aware of these complicating outcomes in order to alleviate these complications.^7^^,^^8^

Perioperative management and surgical techniques are of paramount importance in determining patient’s outcome and post-operative complications in ICU. However, there is a paucity of clinical data on the magnitude of risks associated with post-operative complications.^9^ In spite of many publications in the field, research is still needed to determine perioperative factors affecting patient’s outcome. Literature is scarce on factors affecting blood loss; intensive care unit (ICU) and hospital stay in these patients.^9^ In this study, we aimed to determine peri- and post-operative risk factors, which affect the outcome of craniosynostosis surgery.

## MATERIALS AND METHODS

The study was reviewed and approved by the university review board and hospital ethics committee and been performed in accordance with the ethical standards laid down in an appropriate version of the 2000 Declaration of Helsinki. Information about trial was given comprehensively both orally and in written form to the parents. All parents gave their written informed consents prior to their inclusion in the study according to university hospital ethics board committee.

In a retrospective study, 178 pediatric patients with craniosynostosis who underwent primary reconstruction for craniosynostosis (cranioplasty) from 2011 to 2012 were included in the study. Inclusion criteria was patients operated for non-syndromic craniosynostosis. Exclusion criteria was patients with life threatening underlying disease such as metabolic disorders, Cariac diseases and hypothyroidism and meningitis. The same team of surgeons and anesthesiologist were responsible for all the cases in our study. Preoperative consultations were comprehensively provided to all patients. Preoperative clinical evaluation including a detailed family history, genetic testing, medical and psychiatric counselling the family were provided to the patients. 

The neurologic evaluations to determine the functional abilities and to identify increased intracranial pressure (ICP) among other complicating factors were also performed. A careful respiratory exam was performed in children with significant mid-facial retrusion, to assess compromised airway. Additional evaluations by otolaryngologist, and ophthalmologist were also performed as preoperative consultations. For those requiring extensive reconstruction, anesthesiologist and pediatric intensivists worked accordingly. Blood product transfusions and all blood cross match was estimated and recorded.

All patients’ demographic characteristics including age, weight, and sex were extracted from patients file. Perioperative period was from start of anesthesia to the entrance to recovery. Post-operation period was from entering recovery room until discharge from hospital. Data including duration of surgery, anesthesia time, hemodynamics, and amount of blood loss were recorded. Total amount of bleeding was estimated based on ml of blood in suction tank and gauzes in operation room (each immersed gauze considered as 15 ml bleeding) based on the anaesthesiologist evaluation at the end of surgery.

All post-operative complications following neurosurgical procedures including fever in ICU, level of consciousness, re-intubation, and patient’s blood, urine, and other cultures were also recorded and their association with the main outcomes (length of ICU stay) were analyzed. Infection was described as one of patient’s cultures in ICU such as blood, urine, cerebral blood fluid (CSF) and stool cultures were positive. Pre- and post-operative photographs were taken to record and document progress through all phases of treatment. We considered length of ICU stay as primary outcome and length of hospital stay as secondary outcomes.

Statistical calculations were conducted using SPSS (Version 18, Chicago, IL, USA). The parametric variables were presented as mean±SD and were analyzed by student t-test or ANOVA and Pearson correlation test as appropriate. Statistical analysis was performed using Chi-Square or Mann-Whitney U-test and Spearman correlation coefficients for non-parametric samples. *p*<0.05 was considered as statistically significant. Sample size was estimated using sample size calculator software with 95% confidence interval and *p*<0.05.

## RESULTS

One hundred seventy eight infants were included in this study. There were 94 (53%) female and 84 (47%) male patients. Patient’s age ranged from 1 month to 10 years, mean 12.25±6.45 month and weights ranged from 5 to 30 kg, mean 11.12±7.5 Kg. Duration of surgery ranged from 285 minutes to 544 minutes mean 442.6±92.3 minutes (Table 1). Average length of hospitalization was 7.59±3.47 days, with 3.23±1.45 days in the intensive care unit and 4.23±1.22 days on the floor. Mean blood loss (mean±SD) in various operative techniques in patients with craniosynostosis was not significantly different between various types of surgeries (*p*>0.05). Duration of post-operative ICU stay was significantly less than 4 days in various types of surgeries (*p*<0.05).

**Table 1:** Demographic characterization of craiosynostosis patients classified based on their duration of ICU stay (mean±SD

**ICU stay**	**<4 days**	**>=4 days**	***p*** ** value**
Number of patients	114	64	-
Age (years)	2.35±1.66	7.76±3.85	0.01
Weight (kg)	5.92±5.36	5.95±4.68	0.22
Sex (male%)	48	52	0.064
Duration of surgery	221±88.2	238±95.5	0.077
Perioperation MAP	87±31	91±29	0.27

MAP: Mean arterial pressure

**Fig. 1:**
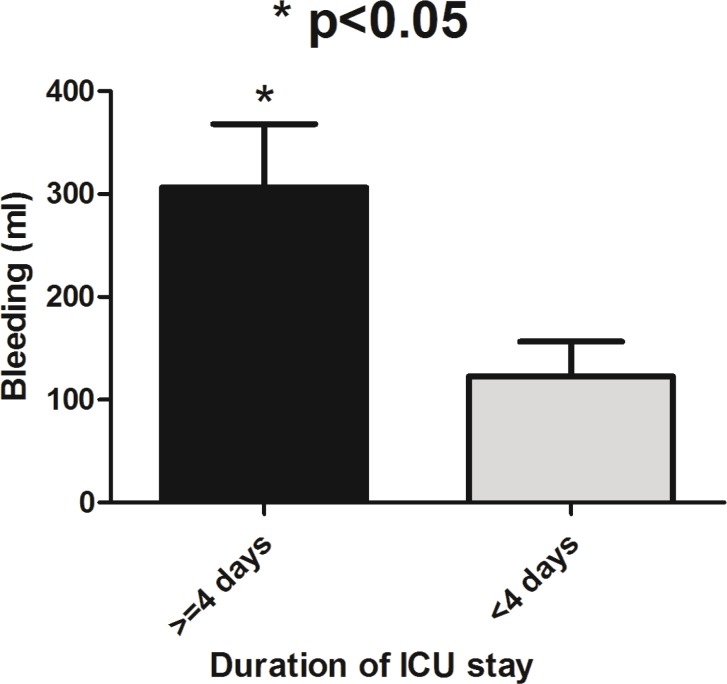
Perioperative bleeding categorized base on duration of post-operative stay in patients with craniosynostosis

**Fig. 2:**
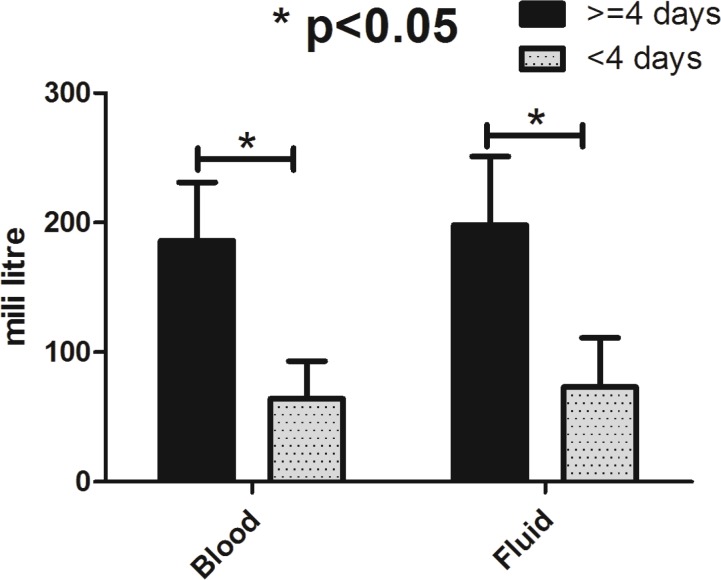
Perioperative transfused blood and infused fluids categorized based on the duration of post-operative stay in patients with craniosynostosis

Mean blood loss during surgery was 320±29.2 ml ranged from 80-400 ml. Perioperative bleeding was significantly higher in patients whose duration of ICU stay was longer than four days compare to the group of less than four days (*p*=0.026) (Figure 1). As mentioned earlier, blood loss during surgery was not different between various operation types (*p*>0.05). Perioperative transfused blood (*p*=0.01) and infused fluids (*p*=0.02) were significantly higher in patients whose duration of ICU stay was longer than 4 days compare to the group of less than 4 days (Figure 2). 

**Fig. 3:**
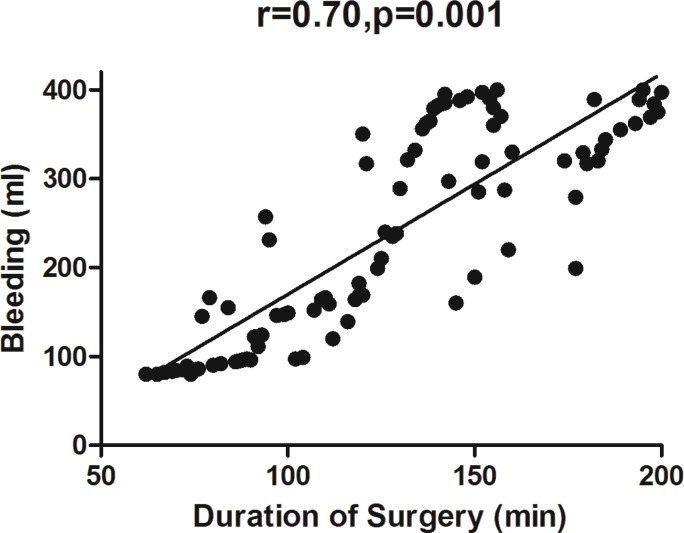
Correlation ratio between duration of surgery and amount of bleeding (ml

**Fig. 4:**
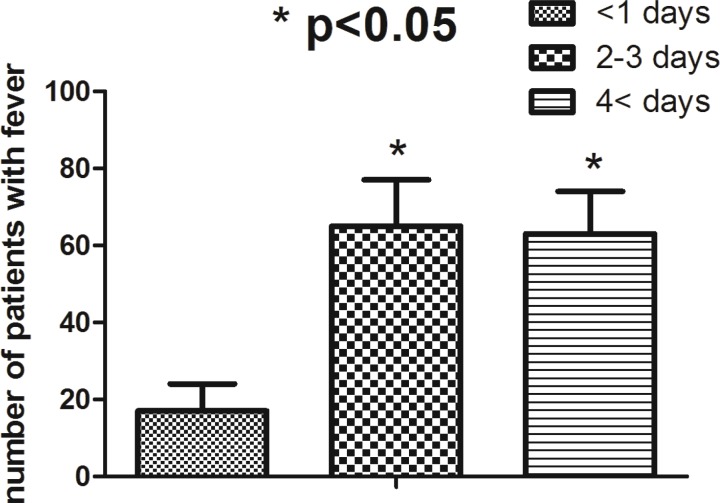
Number of patients with fever categorized base on duration of post-operative stay in patients with craniosynostosis

Amount of bleeding was significantly correlated to the duration of surgery (r=0.70, *p*=0.001) (Figure 3) but not to the patient’s age (r=0.23, *p*=0.44). Only 11 patients (6%) showed sign of hypovolemia (including increase in heart rate and decrease in blood pressure, decrease in skin turgor) in post-operative period, but 34 patients needed blood products (18%). Post-operative fever was presented in 101 (56.7%) of patients while cultures were positive in 15 cases (8.5%). Number of patients with fever was significantly higher in the group of patients whose duration of ICU stay was more than one day (*p*=0.034) (Figure 4).

Factors independently associated with a longer pediatric ICU stay were fever (odds ratio 1.59, 95% confidence interval 1.25-4.32; *p*=0.001), perioperative bleeding (OR=2.25, 95% CI=1.65-3.65; *p*=0.01), age (having surgery after the first 5 years) (OR=1.59, 95%, CI=1.33-3.54, *p*=0.016) and infection (OR=2.17, 95% CI=1.83-7.46; *p*=0.002). Fever (OR=2.24, 95% CI=1.42-4.43; *p*=0.02) was related to the total length of the patient’s hospital stay. Type of surgery was not a risk factor for increase in ICU stay days (OR=1.05, 95% CI=0.54-1.22, *p*=0.15).

## DISCUSSION

Craniosynostosis patients are at risk of several perioperative and post-operative complications, which prolong their ICU stay and delays discharge from hospital and worsen the outcome. Some complications such as infection significantly influenced patients’ outcomes and should be monitored for early diagnosis in post-operative period.^10^ Our study provides understanding to the risk factors of poor outcome in perioperative period. In our study, from the total of 178 patients who underwent cranioplasty, 94.3% of patients had some sort of events requiring longer than one-day intensive care unit stay after craniosynostosis reconstruction surgery.

In our study, the type of operation and craniosynostosis did not influence duration of ICU stay. This assertion is made with the proviso, which the type of operation is supposed to have significant effect on intraoperative bleeding amount.^10^^-^^12^ For instance, trigonocephaly was the most common type of operation in our study, the same as some other reports. One way or other, trigonocephaly or other types were not different in their post-operation outcomes of our patients. Altogether, apparently the degree of expertise of surgeon is supposedly more important than type of surgery.^13^^-^^17^

A patient’s risk of prolonged stay in ICU could be well predicted by large perioperative blood loss and fluid requirements. Our data showed that perioperative blood loss occurs almost continuously and accounts for most poor outcome (prolongation of ICU stay). In fact, bleeding and transfusion is a major predictor of decrease in oxygen carrying capacity and hypovolemia. Our results showed that many of these patients did not have any sign of hypovolemia (94%), but a few still needed blood products (34%). The leading cause of mortality is represented by hemorrhagic derangements after high intraoperative blood loss in pediatric patients.^18^

Vigilant attention to accurate blood replacement, restoration of euvolemia, detection and correction of related bleeding improves outcome of these patients. Older age was a significant risk factor for poor outcome in our study consistent with some previous researches.^19^ Probably the older patients consume more blood products at intraoperative period and at the immediate postoperative period in the PICU compared to younger ones. Besides, duration of ICU stay was significantly correlated to patient’s age in our study. Post-operative morbidities from increased use of blood products can be minimized if cranial vault remodelling is completed at a younger age in patients with primary CSS.^20^

Interestingly, in one study; the only determinant associated with prolonged ICU stay significantly was to have surgery in the first 5 years, which is in contrast to our results.^21^ Overall, it seems if the surgery is performed earlier, the outcomes would be better than postponing the surgical reconstruction. Fever in ICU was significantly associated with longer duration of stay, which was our primary outcome. Interestingly, cultures were positive in only few cases (8.5%), but fever was presented in 101 (56.7%) of patients. This seemingly low positive culture must be viewed in the light of the fact that many patients received prophylactic antibiotics (96% of patients) or the fever may be of unknown origin or a complication of blood products infusion. PICU length of stay is determined in part by postoperative pyrexia and it can be reduced if extensive evaluations of post-operative fever are performed.^22^ Therefore, fever of unknown origin (fever with negative cultures), which is widely reported as a common post-operative morbidity in most series occurred in 48% of our patients.^23^^,^^24^

Craniosynostosis patients who have corrective surgery require post-operation intensive care. Perioperative management factors such as amount of bleeding could have direct effect on their outcomes and length of stay in ICU. Therefore, use of advanced techniques by an expert surgeon for cranioplasty and meticulous observation and perioperative management by anaesthesiologist could significantly improve outcome. 

## CONFLICT OF INTEREST

The authors declare no conflict of interest.
